# A systematic review of the effectiveness of alternative cadres in community based rehabilitation

**DOI:** 10.1186/1478-4491-10-20

**Published:** 2012-08-13

**Authors:** Hasheem Mannan, Camille Boostrom, Malcolm MacLachlan, Eilish McAuliffe, Chapal Khasnabis, Neeru Gupta

**Affiliations:** 1 Centre for Global Health & School of Psychology, Trinity College Dublin, Dublin, Ireland; 2 Disability and Rehabilitation Team, World Health Organization, Geneva, Switzerland; 3 Health Workforce Information and Governance, World Health Organization, Geneva, Switzerland; 4 Centre for Global Health and School of Medicine, Trinity College Dublin, Dublin, Ireland

**Keywords:** Health workers, Alternative cadres, Community based rehabilitation, Systematic review

## Abstract

**Background:**

The Millennium Development Goals (MDGs) aim to improve population health and the quality and dignity of people’s lives, but their achievement is constrained by the crisis in human resources for health. An important potential contribution towards achieving the MDGs for persons with disabilities will be the newly developed Guidelines for Community Based Rehabilitation (CBR), launched in 2010. Given the global shortage of medical and nursing personnel and highly skilled rehabilitation practitioners, effective implementation of the CBR guidelines will require additional health workers, with improved distribution and a new skill set, allowing them to work across the health, education, livelihoods, social, and development sectors.

**Methods:**

We conducted a systematic review to evaluate existing evidence regarding the effectiveness of alternative cadres working in CBR in low and middle income countries. We searched the following databases: PUBMED, LILACS, SCIE, ISMEAR, WHOLIS, AFRICAN MED IND. We also searched the online archive of the Asia Pacific Disability Rehabilitation Journal (available from 2002 to 2010), which was not covered by any of the other databases. There was no limit set on inclusion with regard to how recent a publication was in the general search.

**Results:**

The search yielded 235 abstracts, only 6 of which addressed CBR through some type of evaluative component. Three of the studies explored the effects of CBR interventions, mainly related to physical disabilities, while three explored issues concerned with the work performance of rehabilitation workers. Altogether the studies covered four different countries.

**Conclusion:**

All six studies related to specific service delivery in local contexts, using outcome measures that were not comparable across studies. We do not, therefore, feel that the current results provide adequate methodology or evidence for reliably generalizing their results. Due to the dearth of evidence regarding the effectiveness of alternative cadres in CBR, systematic research is needed on the training, performance and impacts of rehabilitation workers, including their capability of working across sectors and engaging with and making use of health systems research.

## Introduction

**Figure 1:**
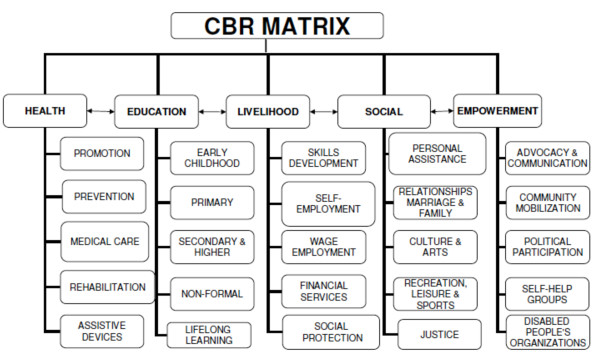
** Community Based Rehabilitation Matrix (World Health Organization:*Community-based Rehabilitation: CBR Guidelines***. Geneva: World Health Organization; 2010).

The Millennium Development Goals (MDGs) are a set of internationally agreed targets on which national, regional and international development initiatives have prioritized their activities. The MDGs have a broad remit, including improving population health and the quality and dignity of people’s lives; but their achievement is constrained by the crisis in human resources for health (HRH) [1]. Fifty-seven countries, many in sub-Saharan Africa, have fewer than 23 medical and nursing professionals per 10 000 population, which is the minimum number estimated as necessary to deliver basic health services to achieve the health-related MDGs [2]. An important potential contribution towards achieving the MDGs for persons with disabilities and other vulnerable groups will be the newly developed Guidelines for Community Based Rehabilitation (CBR), jointly launched in 2010 by the World Health Organization (WHO), UNESCO, the International Labour Organization, and the International Disability and Development Consortium (a consortium of international civil society organizations) [3]. More than 150 experts from across the globe contributed to the draft guidelines, which are being field-tested in 25 countries [4]. The guidelines have five major components: health, education, livelihood, social, and empowerment (see Figure 1). Beyond the five components, the guidelines also focus on the management of special scenarios including CBR and HIV/AIDS, CBR and leprosy, CBR and mental health, and CBR in crisis situations. However, research on CBR to date has been based on a broad range of interpretations of what rationale, theories and practices constitute CBR. The development of the new generic Guidelines for CBR make a review of what we have learnt from traditional practices, both timely and apposite.

The newly developed guidelines offer a path to achieving the MDGs for persons with disabilities and to the realization of the United Nations (UN) Convention on the Rights of Persons with Disability. The World Report on Disability (2011) recognizes that the estimated one billion people with disabilities constitute the world’s largest minority [5], of which 80% live in low income countries [6]. Given the global shortage of medical and nursing personnel [7] as well as highly skilled rehabilitation practitioners [8], effective implementation of the CBR guidelines will require additional health workers, with improved distribution and a new skill set, allowing them to work across the health, education, livelihoods, social, and development sectors. The World Report on Disability and Rehabilitation has given increased impetus to addressing the health needs and health rights of people with disabilities, particularly in low-income countries [9]. Within these countries, it has been argued, scaling up production and deployment of new health workers should be planned and implemented in consideration of service demands by targeting the staff skills that are needed, rather than types of staff [10]. The education and training of a broad-skilled generalist cadre in CBR would be one way to provide the necessary human resources to implement the guidelines.

The CBR guidelines encourage the development of a new curriculum through which to train rehabilitation workers. This curriculum must be interdisciplinary in order to address all five components of the CBR guidelines. The development of the curriculum also must take into account lessons learnt from curricula developed for other alternative cadres. The term “alternative cadre”, in the context of healthcare workers, usually refers to people provided with more focused and shorter training than that normally received by health professions adopting a conventional “Western”-style professional training model, and who undertake equivalent tasks. In particular, the curriculum of schools that train conventional “Western”-style health professionals in Africa have been too focused on the health problems and needs of industrialized countries [11]. Furthermore, the “agenda” for low income country CBR programmes must reflect local needs and resources, as opposed to being determined by often overly dominant aid organizations and institutions from wealthier countries [12]. For instance, the training of rehabilitation workers in universities and training institutions in their own countries would provide an opportunity to develop capacity in such institutions. The curriculum and training programmes will need to be reviewed regularly to ensure continued relevance [13], a process to which those outside low-income countries could contribute. Moreover, the development of regional research centres and networks, with support from international organizations, would allow for the transfer of technical knowledge and skills regarding CBR. This is directly in line with article 32 of the UN’s Convention on the Rights of Persons with Disabilities, which called for international cooperation to facilitate exchange between countries and institutions [14].

There is now good evidence for the clinical efficacy and economic value of alternative cadres in delivering specific healthcare interventions, particularly in maternal health [15-18] and child health [19,20]. However, the situation regarding the use of alternative cadres in CBR is unclear. WHO has advocated for scaling up production and deployment of health workers trained in CBR for several decades [21], and pushing this effort forward now will be a vital part of the implementation of the CBR guidelines. We, therefore, sought to evaluate evidence for the effectiveness of alternative cadres working in CBR in low and middle income countries.

## Methods

We searched the following databases: PUBMED, LILACS, SCIE, ISMEAR, WHOLIS, AFRICAN MED IND. We also searched the online archive of the Asia Pacific Disability Rehabilitation Journal (available from 2002 to 2010), which was not covered by any of the other databases. There was no limit set on inclusion with regard to how recent a publication was in the general search.

We defined “alternative cadre” as health service personnel with more focused and shorter training than that normally received by established (“Western”-styled) clinical professionals. Such staff are referred to by a wide variety of terms within and across countries and over time. In our search we included a broad range of search terms in different languages for “alternative cadre”, including “mid-level providers” and “non-physician clinicians” (see Table 1 for full list of search terms). We combined these search terms with “Community Based Rehabilitation” and synonyms of “low or middle income countries”, including “developing countries” and “Third World”.

**Table 1:** Systematic review search terms for alternative cadre

**Search terms for “alternative cadre”**	
**Agent (adj community, alternative, lay, village, lady, peripheral, low level, mid level, non professional, family, allied)**	**Medical officer (adj community, alternative, lay village, lady, peripheral, low level, mid level, non professional, family, allied)**
**Assistant (adj alternative, adj community, alternative, lay, village, lady, peripheral, low level, mid level, non professional, family, community, clinical, rural health surveillance)**	**Monitora**
**Associate (physician, child health)**	**Mother coordinator**
**Barefoot doctor**	**Non physician clinician**
**Brigadista**	**Nutrition worker**
**Cadre (alternative, lay, village, lady, peripheral, low level, mid level, non professional, family, aide, agent, provider, practitioner, personnel, community, assistant)**	**Practitioner (adj community, alternative, lay, village, lady peripheral, low level, mid level, non professional, family, allied)**
**Chijiao yisheng**	**Promotora**
**Clinical officer**	**Provider (adj community, alternative, lay, village, lady, peripheral, low level, mid level, non professional, family, allied)**
**Colaborador voluntario**	**Radaat**
**Community health aide**	**Rehabilitation (adj worker, Facilitator)**
**Community health worker**	**Resource person**
**Daya(s)**	**Rural health motivator**
**Drug-kit manager**	**Salud (agente, voluntario, tecnicos, promotores)**
**Feldsher**	**Saude (adj agente, promot)ores**
**Health aide**	**Sevika**
**Health aide (adj community, alternative, lay, village, lady, peripheral, low level, mid level, non professional, family, allied)**	**Shasto karmis**
**Health extension worker**	**Shasto shebika**
**Health helper**	**Sub doctor**
**Health personnel**	**Surgical technician**
**Health volunteer**	**Tecnicos de surgia**
**Health worker**	**Traditional (adj birth attendant, midwife, midwives)**
**Health worker (adj community, alternative, lay, village, lady, peripheral, low level, mid level, non professional, family ,allied)**	**Visitor (adj home health, follow-up, lady, health)**
**Kader**	**Voluntary workers**
**Medex**	

While we were particularly interested in interventions that would help us evaluate CBR, we did not enter “intervention” or “evaluation” or synonyms in the search, as we were concerned not to exclude relevant studies that had not included such terminology but might still be cogent.

In order to be eligible for inclusion, a published study had to take place in a resource poor setting, refer to community based rehabilitation, and involve alternative cadres of health workers. It also had to include an evaluative component, that is, beyond a simple discussion of policy issues or description of the implementation of the intervention. The eligible abstracts were reviewed by two individuals, who independently concluded that the same articles met the eligibility criteria.

## Results

The search yielded 235 abstracts. Only six of these addressed CBR through some type of evaluative component. Three explored the effects of CBR interventions, mainly related to physical disabilities, while three explored issues concerned with the work performance of rehabilitation workers. Altogether the studies covered four different countries.

### Intervention effects

Op Heiji et al. [22] examined the experiences of caregivers of children with disabilities (CWDs) in accessing health services in Jamaica and the attitudes of health care workers towards CWDs and their caregivers. A total of 26 caregivers were interviewed, selected from 147 clients participating in the intervention, and 113 health care workers, selected from staff working in health facilities in the intervention region, completed a questionnaire,. Of these 113 health care workers, 17 were community health aides (CHAs), a term included in our search strategy (see Table 1). The study identified poor communication between health workers and caregivers as a major factor in the high level of default among the CWDs. In particular, poor communication led to unrealistic expectations by the caregivers regarding the outcomes of the provided services. While 90% of the CHAs thought that they had made an impact on the caregivers' “beliefs”, 35% believed they had received inadequate training. The study called for improvements in the information provided to caregivers, the communication between caregivers and health workers, and in the training of health workers regarding the management of disabilities.

Vijayakumar et al. [23] focused on service provision to incurably blind people in rural India. An initial sample of 460 984 persons were surveyed via door-to-door ocular screening by trained workers, and persons identified as blind were then categorized as either curable or incurable by an ophthalmologist. The intervention provided CBR to 268 incurably blind people, comprising 67% of an identified 400 individuals who were offered the service. Although the study found that the social and economic rehabilitation services were beneficial to the incurably blind, it identified a need to understand better the barriers to providing such services in order to increase service utilization. Notably, a small proportion of female subjects refused rehabilitative services due to the lack of a female health worker available to assist them. This was, in part, because of difficulties encountered in retaining female health workers, as they were more likely to drop out of the programme due to the inconvenience of traveling to villages outside their own. Overall, the study concluded that CBR may be a viable alternative to the tertiary care approach prevalent in resource poor settings in India in providing services to the incurably blind. Specifically, the authors noted that coverage of rehabilitative services could be increased through training field workers in the rehabilitation of persons with disabilities.

In another case from rural India, Sekaran et al. [24] reported on the reintegration of people with spinal-cord injuries. The sample comprised individuals with spinal-cord injuries living in rural areas who were admitted to the Physical Medicine and Rehabilitation Department of St Johns Medical College Hospital and who were rehabilitated to their functional level based on level of injury. A total of 35 subjects participated. The study was conducted through a standardized questionnaire, and environmental and home assessments were carried out during follow-up home visits over a 12 month period. The study showed a decline in reintegration, although the effects of the community-based rehabilitation workers could not be disaggregated from other health workers. The study concluded that mobility and co-morbidities were the most influential limiting factors regarding the ability of participants to reintegrate with their community. In 92% of the participants’ homes, at least one architectural barrier was present, and 54% had significant limitations in space in their house, which negatively impacted their mobility. Of the 35 subjects, 71.8% had at least one comorbidity which affected their societal participation. Specifically, pressure sores (30%), urinary tract infections (32%), spasticity (26%) and obesity (12%) were the most common morbidities which limited both participation in society and mobility. The findings also suggested that individuals with more severe neurological injury resulting from spinal cord injury and those who were older had decreased levels of community reintegration. This study focused strongly on the clinical aspects of the intervention, and did not directly evaluate the impacts of the community-based rehabilitation workers involved.

Each of the above studies evaluated interventions provided by alternative cadres, rather than focusing on the alternative cadre per se, as the means of delivering the intervention.

### Work performance

Lysack and Krefting [25] explored the factors related to motivation for volunteerism among CBR workers in Indonesia. The study focused on a CBR intervention covering 10 rural communities of 4000 to 5000 persons each, with 50 to 60 persons with disability in each community. Just over 1000 rehabilitation workers were trained through the intervention. Questionnaires were completed by 30 rehabilitation workers (selected via an opportunistic sample). Twelve focus group discussions were conducted with 20 to 30 informants in each group (comprised primarily of rehabilitation workers but also including village leaders, families of disabled persons, and government health officials), and key informant interviews were conducted with 19 rehabilitation workers. The study found that volunteer cadres performed many and varied duties, facing considerable challenges in implementing CBR activities. The authors emphasized the importance of understanding the work from the perspective of those providing services, in addition to those receiving services. They also identified the importance of incentives in determining the motivation and ultimate performance of volunteer cadres. In particular, the study noted several factors which support volunteer programmes, including the existence of cultural structures which prioritize volunteerism, religious values emphasizing serving others, and substantial numbers of people who have few alternative opportunities for training and employment.

Lorenzo [26] conducted qualitative research in South Africa with community rehabilitation workers (CRWs) who had completed a two-year certificate course. The study also included supervisors of CRWs and community members receiving care from CRWs. The research aimed to identify areas in which CRWs needed additional skills, and four key areas were prioritized: advocacy and public education, social work, community development and organizational development. The study was conducted through the nominal group technique and focus group discussions with 8 CRWs and their 5 supervisors, as well as focus group discussions with people with disabilities and their family members (45 people total), from villages covered by the CRWs. People with disabilities, the CRWs themselves, and their supervisors all identified the need for continuing education for CRWs. The author called for continuing education programmes that emphasize capacity building via community-based structures, such as community colleges.

Focusing on the same two-year training programme in CBR in South Africa, Dolan et al. [27] suggested that while the programme had imparted useful skills to CRWs, questions remained as to the adequacy of its coverage overall and among people with different types of disability. The study population included all current and former clients of rehabilitation workers trained through the programme. From the population of 383 clients, a random sample of one former client and four current clients was taken from each rehabilitation worker’s case load, so that the total client sample numbered 40 cases. All clients were then interviewed individually using a structured interview schedule. The findings suggested that rehabilitation workers had a significant impact on the reduction of the functional limitations of the persons with disabilities in their care and contributed to improving their daily living activities. Of those who received care, 60% regarded their increased mobility as the most important benefit of working with a CRW. Additionally, the rehabilitation workers helped to increase their clients’ self esteem and their reintegration into community and family life.

## Discussion

Our systematic literature review focused on alternative cadres of CBR workers in low and middle income countries. Although our interest was particularly in evaluative research, we deliberately withheld this term from the search, in case it overlooked some eligible studies that were evaluative but did not use the term. Despite this inclusive approach, the search yielded 235 abstracts, yet only 6 of these met the inclusion criteria. Among the ineligible studies, most were related to providing care to vulnerable populations in the community, provided by allied health professionals (primarily nurses and physiotherapists) as opposed to alternative cadres of health workers (for instance, “community rehabilitation workers” or “rehabilitation workers”, as described above). Very few studies were concerned with intervention effects and with the work performance of alternative cadres of CBR workers. Most studies had small-to-modest sample sizes and none were nationally representative.

All six studies that met the inclusion criteria related to specific service delivery in local contexts, using outcome measures that were not comparable across studies. While the review does indicate two broad thematic areas of interest – cadre performance and intervention effectiveness – these cannot be effectively combined, such that alternative cadres are evaluated against other more conventional cadres of health workers. Therefore, we feel that the current results do not provide adequate methodology or evidence for reliably being able to generalize their results. Systematic research across varying contexts, processes and programme content is needed [28]. Social, economic, political and cultural contexts; differing health and delivery processes and systems (including the configuration of health services, supervisory support, the role of international aid, government, civil society and other stakeholders) and comparing the content of different training programmes and types of intervention, are some of the important variables for addressing the challenges of both HRH and CBR.

As our systematic review has demonstrated, there is a dearth of evidence regarding the effectiveness of alternative cadres in CBR. However, ample research has identified the effectiveness of alternative cadres in other primary care areas. Previous reviews of the evidence regarding the effectiveness of community health workers (CHWs) have found strong evidence that CHWs can contribute to improvements in uptake of health interventions and health outcomes, including immunization uptake in children, reducing childhood morbidity and mortality, promoting breastfeeding, and improving tuberculosis treatment outcomes [19,29]. But in order for CHWs to be effective they need to be carefully selected, appropriately trained, and continuously supported. Fulton et al. [30] examined the evidence for the effectiveness of task shifting to alternative cadres in resource poor settings, and found it to be a promising policy option in improving the delivery of health services. The authors called specifically for further research examining the development of new cadres of health workers.

The concept of “new” cadres is not itself new. A wide variety of mid-level workers have been successfully providing health care in various countries and contexts for the past 100 years, especially in underserved communities [13]. However, WHO and other international agencies have identified the need for rigorous research to fill knowledge gaps regarding the impacts of mid-level cadres. Specifically, the WHO recommends that data on alternative cadres be routinely collected within human resource information systems [31] and that studies be carried out on the impacts of alternative cadres on health care delivery and outcomes [13]. The Joint Learning Initiative [11] called for operations research as well as monitoring and evaluation focusing on the impact of alternative cadres. In a review of the evidence regarding community health workers, Lehmann and Sanders [19] concluded that systematic assessments of CHW programmes and activities are needed, which must include scientific evaluations and analyses.

The development of alternative cadres of rehabilitation workers with a new skill set, who will work to implement the CBR guidelines on the ground, should take place as part of a response to the aforementioned calls for rigorous research on alternative cadres

CBR interventions and research take place primarily in low and middle income countries, where the majority of persons with disabilities live, and this would provide an important opportunity to develop research capacity in settings where it is greatly needed. While CBR workers and community development workers may have over-lapping roles in some cases, the CBR worker’s role is more focused on the integration and empowerment of people with disabilities. However, it is arguable that people with disabilities represent but one group of marginalized people and that community development should strive towards greater social inclusion for all [32], including a more inclusive approach to health per se [33]. If this is to be the case then a clearer understanding of how these two roles can cooperate, or indeed, how in some cases they could become integrated, needs to be developed.

The implementation of the new guidelines also needs to closely engage civil society organizations, which have been at the forefront of CBR interventions to date, in order to both learn from their experience and to ensure a collaborative process. Additionally, research on access by persons with disabilities to health care services, along with measures of disability, can be used as a key probe in evaluating equity in health systems [34]. The creation of an evidence base to this effect would allow for comparisons of equity in health systems across countries and regions.

In order to ensure the long term motivation and retention of new cadres of rehabilitation workers, a variety of lessons learnt from general CHW programmes can be applied. Access to continuing education and supportive supervision is vital, and needs to be provided by staff with appropriate experience [13]. Well-managed systems for recognizing performance can increase an individual health worker’s motivation and can enhance the respect and status of the health worker in the community [11]. A health team approach is also important, so that the role of the CHW is clear in relation to the role of other team members and to ensure an appropriate skill mix [13]. Ensuring that rehabilitation workers are trained with an appropriate skill mix will also enable them to work in a multisectoral environment, which is crucial to the success of CBR programmes [35,36].

The successful implementation of the new guidelines for CBR requires the development and deployment of rehabilitation workers who are capable of engaging with and making use of health systems research and of working across sectors that interface with health systems. The creation of new cadres of alternative health workers and the implementation of the guidelines would strongly support efforts to improve the lives of the 650 million persons with disabilities throughout the world [14], and would bring us closer to equitable access to health care.

## Competing interests

The authors declare that they have no competing interests.

## Authors’ contribution

HM, CB, and MM reviewed abstracts for the systematic review and drafted the manuscript. EM, CK, and NG contributed to the manuscript. All authors read and approved the final manuscript.
